# Descriptive outcomes for a cohort of high-frequency psychiatric service users in the Western Cape, South Africa after 10 years

**DOI:** 10.4102/sajpsychiatry.v28i0.1821

**Published:** 2022-05-27

**Authors:** Danell Coetzee, Liezl Koen, Dana Niehaus, Ulla Botha

**Affiliations:** 1 Department of Psychiatry, Faculty of Medicine and Health Sciences, Stellenbosch University, Cape Town, South Africa

**Keywords:** assertive community treatment, deinstitutionalisation, hospitalisation, high-frequency user, admissions, admission days, South Africa

## Abstract

**Background:**

Assertive community treatment (ACT) is an intervention implemented to manage the effects of deinstitutionalisation. South African studies have reported decreased admissions at 12 and 36 months when a modified ACT intervention is compared with standard care. However, costs associated with the intervention have raised the question of its feasibility in developing countries.

**Aim:**

This study aimed to describe the long-term demographic and clinical outcomes of a group of psychiatric high-frequency users (HFUs) included in the first South African ACT study.

**Setting:**

Stikland Psychiatric Hospital, Cape Town, South Africa.

**Methods:**

Data from 55 HFUs participating in the first South African ACT trial, including both the intervention and control groups, were retrospectively reviewed 10 years after the patients’ inclusion.

**Results:**

Of the 55 HFUs initially included, nine remained in the formal ACT programme whilst 16 received standard care over the full 10 years. Five patients died and two were admitted to long-term wards. The mean number of admissions was 3.73 and the mean number of admission days was 261.11 over the 10 years. Twelve patients were never re-admitted; of these, nine came from the original study intervention group.

**Conclusions:**

This was the first study looking at the long-term outcomes of a group of psychiatric HFUs in an under-resourced setting receiving either a modified ACT intervention or standard outpatient care. Reflecting broadly on the group, there were a larger number of patients in the original ACT group who had no re-admissions and a comparatively higher utilisation of available services during the 10-year follow-up period.

## Introduction

Deinstitutionalisation refers to the movement of mental healthcare users from long-term inpatient facilities to community care.^1^ In South Africa, deinstitutionalisation caused significant pressure on mental health services as the drastic reduction in the number of inpatient beds happened without providing sufficient community-based placements and resources to address the needs of mental healthcare users.^2^ Scarcity of beds in psychiatric facilities has led to the emergence of premature discharge policies where patients are discharged before they have fully recovered. This has exacerbated the ‘revolving door phenomenon’ where patients are admitted to hospital repeatedly, staying for only a short time before being prematurely discharged.^3,4^ To address the pressure on inpatient facilities following deinstitutionalisation, a number of outpatient interventions have been implemented.

Assertive community treatment (ACT) is an established team-based outpatient treatment intervention. Patients are assigned to a keyworker who oversees and closely monitors their treatment, with support from a multidisciplinary team. The core features of ACT are small caseloads, access to 24-hour care, a multidisciplinary approach, regular meetings, careful monitoring of medication and individualised care.^5^ Assertive community treatment has been implemented in many countries to both support patients in the community and to reduce readmission rates.^6^

The effectiveness of the ACT intervention has been extensively studied in high-income countries (HICs), with initial studies reporting both a reduction in hospital inpatient days and improved patient satisfaction.^7^ A study performed in the Netherlands on a flexible ACT intervention concluded that the longer the duration of ACT the better the outcomes.^8^ However, there is an ongoing discourse regarding the effectiveness of ACT, given the varying degrees of success reported in the literature. Initial positive outcomes relating to reduction in admission days found in studies from the United States of America in the 1990s could not be replicated in two large United Kingdom (UK) studies.^7^ The UK studies (namely UK700 trial and PRISM trial) found no reduction in hospitalisation rate in groups receiving ACT versus standard care.^9,10,11^ These conflicting findings cast doubt on the notion of the cost-effectiveness of ACT as a psychiatric treatment intervention. To elucidate the factors that could have contributed to poor outcomes, Burns^6^ conducted a meta-analysis which included 64 studies on ACT. The author reported that many studies failed to properly define their control group, with many of the core ACT features implemented as part of standard care. Other studies have also suggested that the positive outcomes of community-based interventions, including ACT, would be more pronounced in settings where standard care is less comprehensive.^3,12,13^

Fewer studies have been carried out on the effectiveness of ACT interventions in lower resourced settings, but South African data are available. To address the pressure on inpatient bed availability, a modified ACT intervention was implemented in the Western Cape, South Africa in 2007, as part of a randomised controlled trial. Patients meeting predefined high-frequency user (HFU) criteria were randomly assigned to either the modified ACT intervention or standard care. The intervention differed from standard ACT treatments by allowing for bigger caseloads and less frequent visits. After-hours services were available via the standard on-call service.^14^ At 1-year follow-up, participants in the modified assertive intervention had spent fewer days in hospital and had lower Positive and Negative Syndrome Scale (PANSS) scores compared with participants receiving standard care.^15^ A longitudinal study reporting on the same participants after three years of treatment found that the modified ACT intervention was able to sustain reductions in inpatient usage over time.^16^

This study aimed to describe the clinical and demographic outcomes of the HFUs in an under-resourced setting included in the initial ACT study, 10 years after inclusion.^15^

## Methods

### Study design

The authors conducted a retrospective review of the group of psychiatric HFUs, 10 years after their inclusion in the first South African ACT trial.^15,16^ Patient data from both the intervention and control groups were reviewed and reported on as a whole.

### Study setting

The study was conducted at Stikland Psychiatric Hospital in Cape Town, South Africa. The hospital provides inpatient and outpatient services to an area housing a population of approximately 1.7 million people.

The ACT team at the hospital consists of a part-time medical officer, social worker, psychiatrist and three full-time chief psychiatric nurses. The ACT team also has access to a psychologist, an occupational therapist, a dual-diagnosis service, a step-up/step-down (SUSD) facility, and a psychosocial rehabilitation-based (PSR) day programme on a referral basis. The modified ACT team at Stikland Hospital differs from the international model by having bigger caseloads (30–35 patients per keyworker) and less frequent home visits (weekly or fortnightly).

It is important to note that after the initial 12-month study period, some patients receiving standard care were included in the ACT service to give more patients the opportunity to benefit from the intervention. The team utilised well-defined referral and discharge criteria, resulting in some patients being discharged from the ACT service during the 10 years. Common reasons for discharge included (1) clinical stability and engagement with community-based standard care services, (2) ongoing poor engagement with the team or refusing home visits and (3) relocating patients. Discharge of stable patients and patients who were not engaging allowed for the service to be available to more patients. However, if previously stable ACT patients relapsed after discharge from the service, they were re-included into the intervention.

### Study sample

This study describes the same group of patients who were part of the ACT study in South Africa.^15,16^ These were all high-frequency psychiatric users diagnosed with schizophrenia or schizoaffective disorder enrolled between January 2007 and March 2010 and subsequently randomised into two groups: one receiving the modified ACT intervention and the other receiving standard care. A total of 55 patients were retrospectively reviewed for their outcomes over 10 years. For a full description of inclusion criteria, see Botha et al.^15^

### Data collection

Data were obtained from patient folders and from the Clinicom Application Manager (a Western Cape hospital database keeping records on patient demographics, outpatient appointments and hospital admissions). Where no recent folders were available, patients were contacted telephonically. All data were collated in a Microsoft Excel spreadsheet. Information was extracted for demographics, number of acute psychiatric hospital admissions, the total days spent in hospital over the 10-year period, admissions to other psychiatric services and adverse events.

### Data analysis

For descriptive analyses, nominal data were summarised as counts and frequencies, whilst numerical data were summarised as means with standard deviation. All analyses were performed using SPSS version 26.0.

### Ethical considerations

Ethical approval for the parent study was obtained from the Health Research Ethics Committee of Stellenbosch University (ref #: N06/07/140). An amendment was approved for this study, requesting a waiver of informed consent based on the retrospective nature of the study. All data were anonymised to ensure privacy and confidentiality of participants’ personal information, with each participant assigned a unique identifier. This study was conducted in accordance with the South African Good Clinical Practice Guidelines^17^ and the Declaration of Helsinki.^18^

### Results

The small sample size and movement of patients between groups did not allow for a statistical comparison, but rather a description of the group in totality. The demographic characteristics of 55 study participants, 10 years after they were included in the first ACT study in South Africa, are summarised in Table 1. Participants were predominantly male (76%) and had a mean (standard deviation [SD]) age of 43 (9.69) years. Most participants lived in an urban area (91%), were single (82%) and spoke Afrikaans as their home language (91%). The overall group had a low level of education, with only 12.7% having completed Grade 12.

**TABLE 1:** Demographic variables for all study participants (*n* = 55).

Variable	*n*	%	Mean	Standard deviation
**Age**	-	-	43	9.69
**Sex**				
Female	13	24	-	-
Male	42	76	-	-
**Area of residence**				
Urban	50	91	-	-
Rural	5	9	-	-
**Marriage status**				
Married	4	7	-	-
Divorced	6	11	-	-
Single	45	82	-	-
**Language**				
English	1	2	-	-
Afrikaans	50	91	-	-
Xhosa	4	7	-	-
**Education**				
Grade 12	7	12.7	-	-
Secondary education excluding Grade 12	28	50.9	-	-
Elementary only	18	32.7	-	-
No formal education	2	3.7	-	-

**FIGURE 1:**
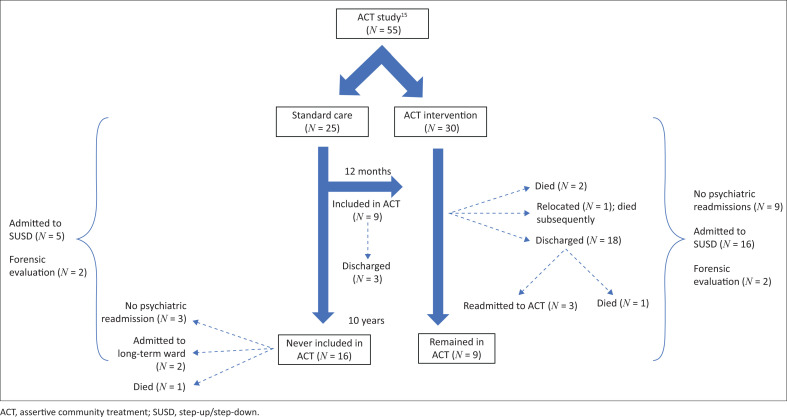
Flow diagram describing patient outcomes over 10 years, included in the ACT study^15^ in South Africa.

Of the 55 study participants retrospectively followed up after 10 years, with no patients lost to follow-up, 30 patients (55%) were randomised to the ACT initial intervention (ACT-i) whilst 25 (45%) received standard outpatient care (Figure 1). Of those participants initially included in ACT-i, all continued to the formal programme (ACT-fp) after 12 months, nine (30%) remained for the full 10 years, two died whilst receiving ACT-fp and one was discharged because of relocation (and subsequently died). The remaining 18 (60%) were discharged from ACT-fp at different time points over the 10 years, of whom one had subsequently died and three were readmitted into ACT-fp. Of the 30 patients originally included in ACT-i, nine (30%) had no psychiatric readmissions. During the 10-year follow-up, 16 (53%) had one or more admissions to a SUSD facility, two had forensic evaluations and none had admissions to long-term wards.

Of the 25 participants originally included in standard outpatient care, nine (36%) were included in ACT-fp during the following 10 years, of which three (12%) were later discharged. A total of 16 (64%) participants remained in standard outpatient care for the full 10 years, of whom three (12%) were never readmitted, two were admitted to long-term wards and one had died. Of those who received standard outpatient care, five (20%) had one or more admissions to a SUSD facility and two had forensic evaluations. Of the overall sample, during the 10-year period, five participants died, one by suicide (age 31), two from cancer at age 39 and 51 and two from unknown causes (aged 51 and 54, respectively).

A description of participants who received ACT intervention and were admitted to acute psychiatric services is summarised in Table 2. The mean (SD) number of days spent in acute hospital services was 261.11 (481.25) days, with some participants not admitted whilst others spent up to 2523 days. There were two outliers who were admitted to long-term wards during the 10-year period and therefore had extended hospitalisation times (> 1500 days).

**TABLE 2:** Number of admission and admission days.

Dimension	Mean	Minimum	Maximum	Standard deviation
Number of admissions	3.73	0	18	3.99
Number of admission days	261.11	0	2523	481.25

## Discussion

To our knowledge, this is the only study to date reporting on 10-year outcomes of high-frequency psychiatric users in an under-resourced setting. Whilst the small sample size and movement of patients between groups did not allow for a statistical comparison, broad comparisons of some outcomes could be reflected on.

Social outcomes from this 10-year longitudinal study correlated with results from the MRC ÆSOP-10 study, which reported poor social outcomes related to marriage, independent living and employment in their large sample of patients with schizophrenia when compared with the general population.^19^ The study concluded that social exclusion after a first psychotic presentation persists for the long term.^19^ These expected social outcomes were more pronounced in our resource-poor setting, where only 12% had a high school education and more than 80% were reported to be single. The majority of the sample lived with family members, a common finding in studies reporting in this setting.^16^ As a result of limited residential placement options, many patients are invariably discharged into the care of their family.

Schizophrenia has been associated with early mortality when compared with the general population.^20^ This early mortality has largely been linked to preventable causes that include trauma, suicide and cardiovascular complications.^20^ Furthermore, side effects of antipsychotics include weight gain that could lead to diabetes, hypertension and dyslipidaemia, which are associated with cardiovascular complications.^21,22^ Patients with chronic mental illness often have a more sedentary lifestyle, which may further contribute to cardiovascular risks.

Sher et al. reported in 2019 that the lifetime suicide rate in patients with schizophrenia is 10%, with provision of comprehensive treatment reported to be the only reliable protective factor.^23^ Interestingly, the 10-year follow-up of the ÆSOP first-episode cohort reported comparatively high mortality rates, specifically those as a result of unnatural causes, with the most common cause of unnatural deaths cited as suicide. Out of an overall sample of 549 participants, 13 (2.4%) had died as a result of suicide.^24^ Considering all of these factors, one might have expected a higher mortality rate in our group of HFU’s. The patient who died of suicide had relocated and was discharged from the ACT service a year before their death. One possible explanation is that the support patients receive whilst in the ACT service, with frequent contacts, review of medication and proactive follow-up, may have reduced the impact of the factors that drive mortality in this group.

Duration of admissions varied, with some patients spending prolonged periods of time admitted to acute psychiatric services during the 10-year period. Two participants remained so unwell that they required extended admission to long-term wards. Long-term admissions are often utilised when patients have persistent symptoms that put them or their carers at risk, despite biological and psychosocial interventions. There were however some patients who did not have any acute admissions during the 10-year period. This may be the result of better adherence and symptom control because of the proactive approach of ACT staff in preventing relapse.

A high utilisation of SUSD admissions for this group of HFUs was also observed. Most admissions were ‘step-down’ admissions from acute units but some were ‘step-up’ admissions as outpatients. One of the roles of the ACT service is to facilitate optimal use of all available resources, which would include making use of ‘step-up’ admissions to avert acute admissions in patients displaying early relapse signs.

An important consideration and limiting factor of this study is the small sample size and that a modified ACT strategy was employed. ACT keyworkers had significantly larger caseloads to oversee (up to 35 compared with 15 in standard ACT models), with less-frequent contacts. Another limiting factor was that some data, such as information about disability grants, was not available for patients who were followed up elsewhere. After-hour services are also overseen by on-call doctors who may not know the ACT patients, and although the ACT team provided telephonic after-hours support to the doctor on-call, unnecessary admissions may have occurred. In addition, the movement between groups during the 10-year period made direct comparison impossible. This was a patient-centred decision to give more patients the opportunity to benefit from the ACT intervention.

## Conclusion

This study provided a unique perspective on the long-term outcomes of high-frequency psychiatric users who were included in the first ACT study in South Africa. Reflecting broadly on the group, those who originally received ACT included a larger number who had no re-admissions and showed a comparatively higher utilisation of available services during the 10-year follow-up period. Future studies should include long-term follow-up of larger sample sizes and consistent control groups to determine the effect of a modified ACT approach on admission rates and secondary outcomes, such as patient satisfaction, engagement with service and treatment adherence.
